# Restrictive cardiomyopathy due to new mutation in the ACTN2 gene: a case report

**DOI:** 10.1093/ehjcr/ytaf421

**Published:** 2025-08-26

**Authors:** Bo Lan, Zhiyu Liu, Jing Bai, Junnan Tang, Jinying Zhang

**Affiliations:** Department of Cardiology, The First Affiliated Hospital of Zhengzhou University, No. 1, Jianshe East Road, Erqi District, Zhengzhou, Henan 450052, China; Henan Province Key Laboratory of Cardiac Injury and Repair, The First Affiliated Hospital of Zhengzhou University, No. 1, Jianshe East Road, Erqi District, Zhengzhou, Henan 450052, China; Henan Province Clinical Research Center for Cardiovascular Diseases, The First Affiliated Hospital of Zhengzhou University, No. 1, Jianshe East Road, Erqi District, Zhengzhou, Henan 450052, China; Tianjian Laboratory of Advanced Biomedical Sciences, Academy of Medical Sciences, Zhengzhou University, 40 Daxue North Road, Erqi District, Zhengzhou, Henan 450052, China; Department of Cardiology, The First Affiliated Hospital of Zhengzhou University, No. 1, Jianshe East Road, Erqi District, Zhengzhou, Henan 450052, China; Henan Province Key Laboratory of Cardiac Injury and Repair, The First Affiliated Hospital of Zhengzhou University, No. 1, Jianshe East Road, Erqi District, Zhengzhou, Henan 450052, China; Henan Province Clinical Research Center for Cardiovascular Diseases, The First Affiliated Hospital of Zhengzhou University, No. 1, Jianshe East Road, Erqi District, Zhengzhou, Henan 450052, China; Tianjian Laboratory of Advanced Biomedical Sciences, Academy of Medical Sciences, Zhengzhou University, 40 Daxue North Road, Erqi District, Zhengzhou, Henan 450052, China; Department of Cardiology, The First Affiliated Hospital of Zhengzhou University, No. 1, Jianshe East Road, Erqi District, Zhengzhou, Henan 450052, China; Henan Province Key Laboratory of Cardiac Injury and Repair, The First Affiliated Hospital of Zhengzhou University, No. 1, Jianshe East Road, Erqi District, Zhengzhou, Henan 450052, China; Henan Province Clinical Research Center for Cardiovascular Diseases, The First Affiliated Hospital of Zhengzhou University, No. 1, Jianshe East Road, Erqi District, Zhengzhou, Henan 450052, China; Tianjian Laboratory of Advanced Biomedical Sciences, Academy of Medical Sciences, Zhengzhou University, 40 Daxue North Road, Erqi District, Zhengzhou, Henan 450052, China; Department of Cardiology, The First Affiliated Hospital of Zhengzhou University, No. 1, Jianshe East Road, Erqi District, Zhengzhou, Henan 450052, China; Henan Province Key Laboratory of Cardiac Injury and Repair, The First Affiliated Hospital of Zhengzhou University, No. 1, Jianshe East Road, Erqi District, Zhengzhou, Henan 450052, China; Henan Province Clinical Research Center for Cardiovascular Diseases, The First Affiliated Hospital of Zhengzhou University, No. 1, Jianshe East Road, Erqi District, Zhengzhou, Henan 450052, China; Tianjian Laboratory of Advanced Biomedical Sciences, Academy of Medical Sciences, Zhengzhou University, 40 Daxue North Road, Erqi District, Zhengzhou, Henan 450052, China; Department of Cardiology, The First Affiliated Hospital of Zhengzhou University, No. 1, Jianshe East Road, Erqi District, Zhengzhou, Henan 450052, China; Henan Province Key Laboratory of Cardiac Injury and Repair, The First Affiliated Hospital of Zhengzhou University, No. 1, Jianshe East Road, Erqi District, Zhengzhou, Henan 450052, China; Henan Province Clinical Research Center for Cardiovascular Diseases, The First Affiliated Hospital of Zhengzhou University, No. 1, Jianshe East Road, Erqi District, Zhengzhou, Henan 450052, China; Tianjian Laboratory of Advanced Biomedical Sciences, Academy of Medical Sciences, Zhengzhou University, 40 Daxue North Road, Erqi District, Zhengzhou, Henan 450052, China

**Keywords:** Case report, Restrictive cardiomyopathy, ACTN2 gene, New mutation

## Abstract

**Background:**

Restrictive cardiomyopathy (RCM) is a relatively rare cardiomyopathy. We report a case of familial restrictive cardiomyopathy confirmed by myocardial biopsy and genetic testing.

**Case summary:**

A 19-year-old male presented with recurrent syncope and cardiac arrest episodes over 1 year. Genetic testing identified a novel heterozygous insertion mutation (c.2489_2490insTTGCT, p.Q830Hfs*73) in the ACTN2 gene, altering a highly conserved amino acid sequence. Immunohistochemical analysis of endomyocardial biopsies revealed significantly elevated ACTN2 protein expression (77.34% positivity, H-Score 132.14). This mutation represents a likely pathogenic variant accounting for sudden cardiac deaths in multiple male family members.

**Discussion:**

Restrictive cardiomyopathy has a rigid, noncompliant left ventricle, and left ventricular systolic function is usually preserved in the early stages of RCM but tends to deteriorate over time. In this case, the only adaptive response that can increase cardiac output is an increase in the heart rate, which may be attenuated in patients with concomitant autonomic dysfunction, thereby increasing the risk of hypotension during exercise. Therefore, hypotension due to decreased left cardiac output may have been the main cause of post-exercise syncope in this case.

In this report, we used whole-exome sequencing to identify a mutation in the ACTN2 gene of this RCM patient. Subsequently, we performed a one-generation validation in his mother and his brother and analysed the correlation between this gene variant and the RCM phenotype. This may be helpful for early identification and diagnosis of RCM.

Learning points

- A novel pathogenic ACTN2 frameshift mutation causes restrictive cardiomyopathy (RCM) by disrupting sarcomere function and increasing abnormal protein accumulation.
- Diastolic dysfunction and autonomic dysregulation drive syncope in RCM despite preserved systolic function.

## Introduction

Restrictive cardiomyopathy (RCM) is a relatively rare cardiomyopathy usually caused by impaired ventricular filling due to increased myocardial stiffness, characterized by incomplete dilatation of the left or right ventricle with diastolic dysfunction.^1^ Common acquired causes include myocarditis, peripartum, and stress induced.^2^ However, it is the hereditary factors that are the most important cause of the onset of RCM.^3^

More than 20 mutated genes associated with RCM have been reported to cause RCM, which is usually inherited in an autosomal dominant manner. Cases with a familial predisposition often present with incomplete episodic autosomal dominant inheritance, usually due to mutations in multiple genes. Currently, RCM is usually diagnosed using imaging tests, such as a typical echocardiogram. In addition, cardiac magnetic resonance imaging (MRI) is also recommended for the diagnosis of RCM. However, the definitive diagnosis of RCM currently still relies on cardiac catheterization or endomyocardial biopsy.

In this report, we identified a shifted-code mutation in the ACTN2 gene in a patient with RCM using whole-exome sequencing, validated it in one generation in her mother and her brother, and analysed the correlation between the gene variant and the phenotype of RCM. This may be helpful for early identification and diagnosis of RCM.

## Summary figure

**Figure:**
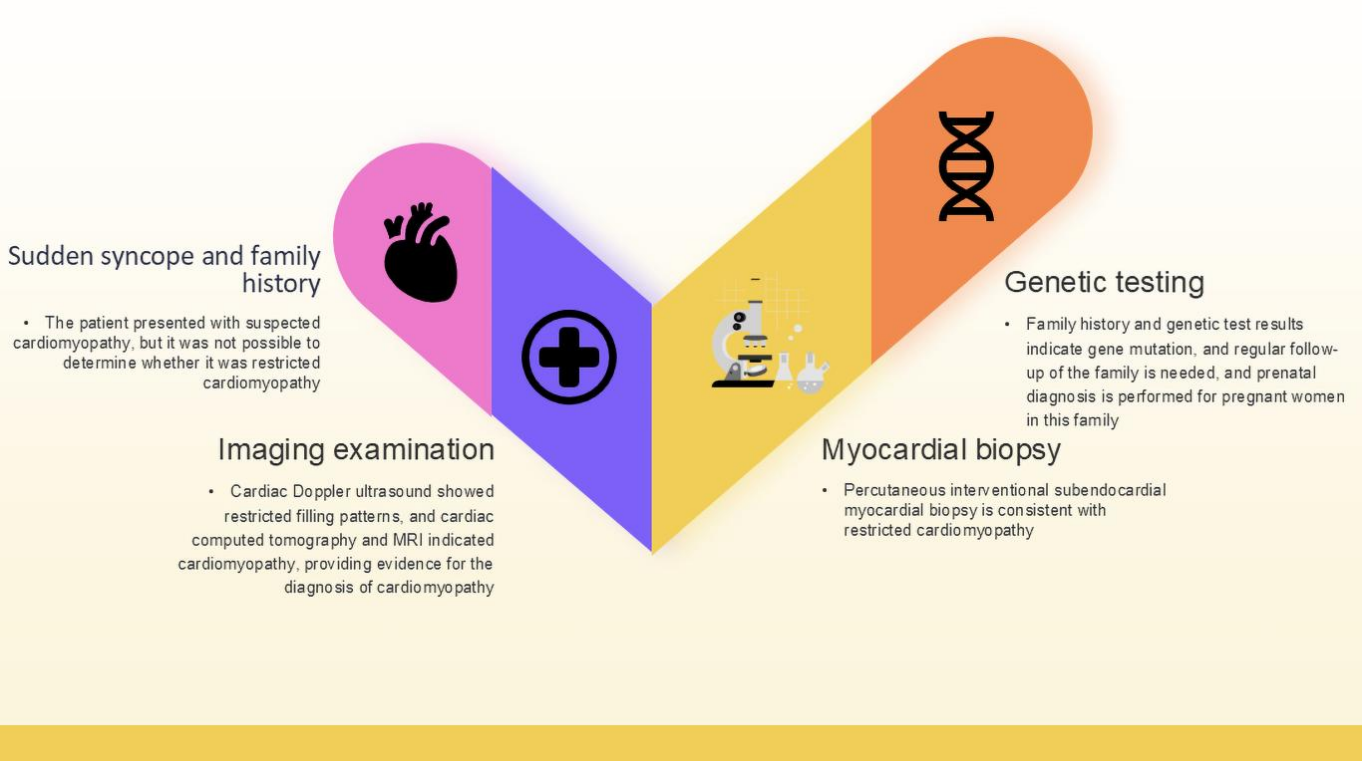


## Case presentation

A 19-year-old male patient suffered a sudden cardiac arrest and syncope after exercise for the first time in January 2022 and regained consciousness after cardiopulmonary resuscitation. The patient had been diagnosed with hypertrophic non-obstructive cardiomyopathy in other hospitals and had been treated with trimetazidine and metoprolol, which were ineffective, and he was hospitalized in our hospital for further diagnosis and treatment and was admitted to the hospital with ‘cardiac arrest and primary cardiomyopathy’. The patient’s father had died of cardiac arrest at the age of 35 years, and his older brother had similar symptoms to the patient, so a familial genetic disease could not be excluded.

The physical examination showed that the patient had enlarged heart boundaries, no murmurs were heard in the auscultation zones of the valves, and there were no pericardial friction sounds. Initial laboratory tests showed a significant elevation of the patient’s B-type natriuretic peptide precursor to 4316 pg/mL, at which point the patient’s cardiac function was Class II according to New York Heart Association classification criteria.

**Figure 1:**
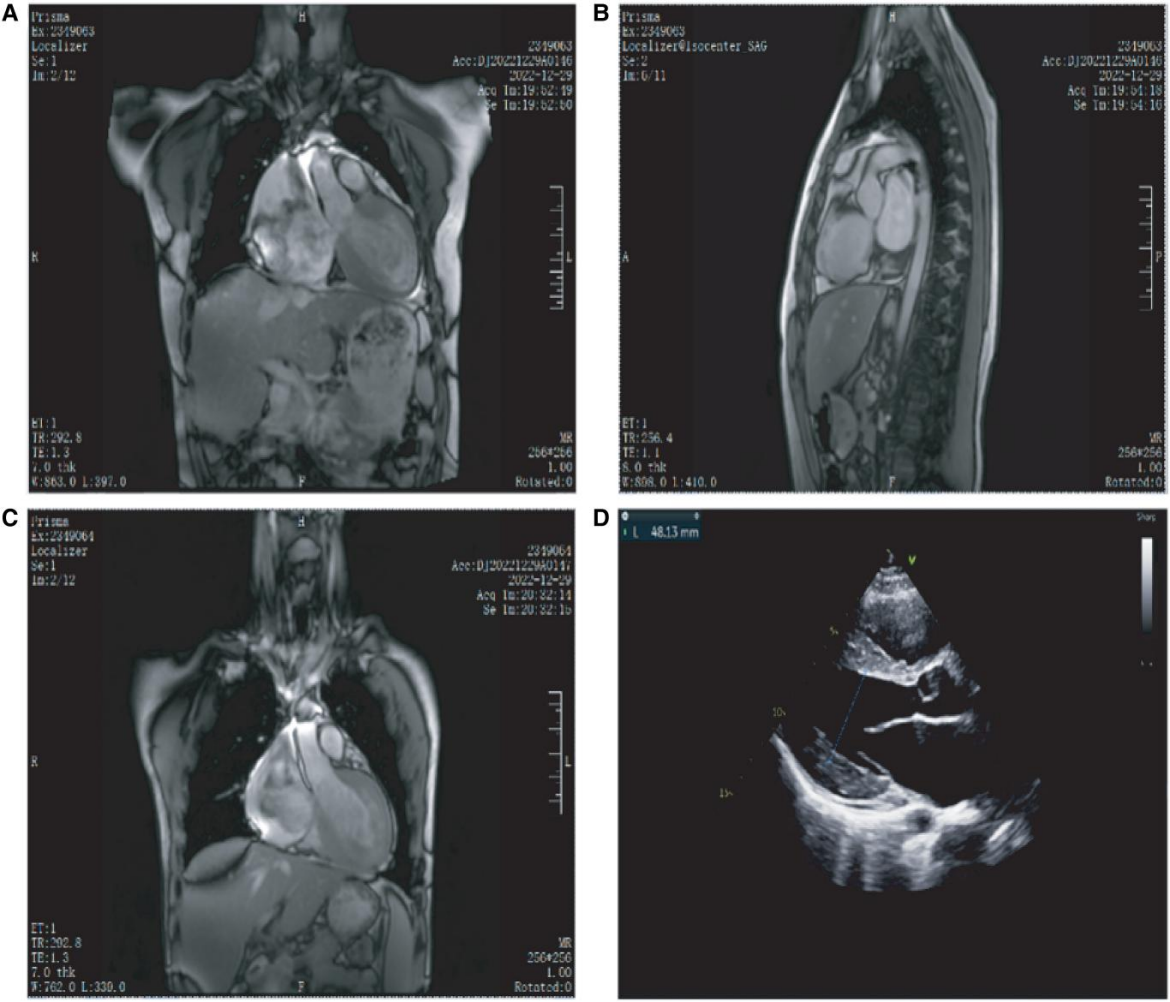
Imaging findings in the proband and his brother. (*A* and *B*) Cardiac MRI findings in the proband. (I) Cardiac MRI findings in the proband’s brother. (*D*) Cardiac ultrasound findings in the proband.

The patient underwent cardiac nuclear magnetic resonance imaging (CMR) 1 year prior to admission, which showed bi-atrial enlargement, predominantly in the right atrium (left atrial internal diameter of ∼41 mm × 83 mm and right atrial internal diameter of ∼73 mm × 91 mm). The left ventricular wall showed uniform thickening (septum 11–16 mm, anterior/lateral walls 12–14 mm) with preserved ejection fraction (52%). Delayed enhancement imaging demonstrated mild linear mid-wall fibrosis in the basal septum. The right ventricle was slightly larger (a transverse diameter of about 55 mm), and the right ventricular outflow tract (29 mm) and the main pulmonary artery were widened (17 mm). Echocardiographic correlation revealed reduced right ventricular function (Tricuspid Annular Plane Systolic Excursion 14 mm) and severe diastolic dysfunction (E-wave to A-wave ratio > 2). The valve structure was normal except for mild mitral and tricuspid insufficiency. The inferior vena cava and hepatic vein were significantly widened (*Figure 1A* and *B*). The CMR results of the proband’s brother showed a similar pattern (*Figure 1C*).

After admission, we improved other imaging tests. Chest computed tomography (CT) suggested an enlarged cardiac shadow with prominent atria and slightly hypodense liver. Cardiac ultrasound was additionally suggestive of a homogeneous thickening of the left ventricular wall compared to the aforementioned magnetic resonance findings, which suggested that the patient might have cardiomyopathy (*Figure 1D*). Holter monitoring revealed sinus bradycardia (mean heart rate 52 b.p.m., range 36–75) with occasional atrial ectopics (35 total), brief pauses (max 2.0 s), and first-degree atrioventricular block, but no sustained ventricular arrhythmias. While these findings could potentially exacerbate haemodynamic compromise in RCM, the absence of high-grade conduction abnormalities or malignant arrhythmias suggests they were unlikely the primary cause of syncope/cardiac arrest. Based on the patient’s clinical manifestations and test results, we initially considered that the patient was suffering from cardiomyopathy affecting cardiac function and thus causing syncope.

In order to clarify the pathological type of the patient’s cardiomyopathy, after excluding contraindications to surgery, we performed percutaneous interventional endomyocardial biopsy under local anaesthesia, using myocardial biopsy forceps to take myocardial specimens from different parts of the myocardium for examination on four successive occasions, and after the procedure, we reviewed the cardiac ultrasound, and the results were unchanged compared with those of the preoperative period.

While waiting for the pathology results of the myocardial biopsy, the patient decided to be discharged from the hospital, and we instructed the patient to take regular out-of-hospital doses of sacubitril valsartan sodium tablets 50 mg twice a day, dapagliflozin tablets 10 mg once a day, furosemide tablets 20 mg once a day, spironolactone tablets 20 mg once a day, and ursodeoxycholic acid tablets 250 mg twice a day.

**Figure 2:**
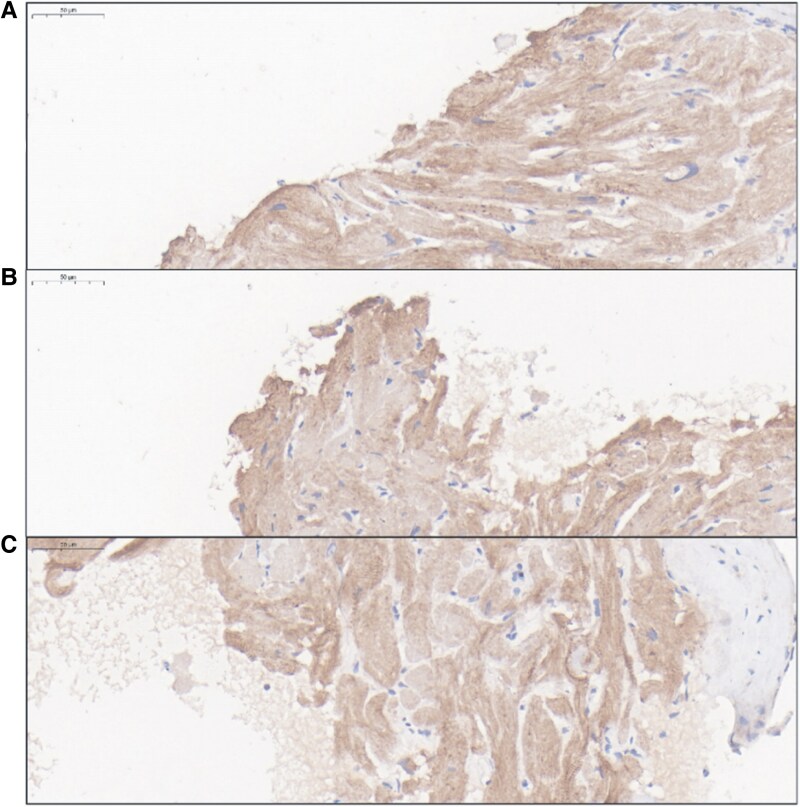
Results of immunohistochemical staining of myocardial tissue specimens.

After waiting for about a week, we received the pathology report card for the myocardial biopsy. Light microscopy revealed (i) cardiomyocyte hypertrophy, (ii) disordered cellular arrangement, and (iii) small foci of interstitial fibrous tissue proliferation, with no inflammatory infiltrates or amyloid deposition. Electron microscopy showed normally arranged myofibrils without dissolution, slightly increased mitochondrial numbers, and no abnormal inclusions. Immunohistochemical staining demonstrated focal dystrophin deficiency with preserved desmin and lysosomal markers (LAMP-2, galactosidase). The protein encoded by the ACTN2 gene was highly expressed in cardiomyocytes, with a positive cell ratio of 77.34% and an H-Score of 132.14 (*Figure 2*). These findings support the diagnosis of restrictive cardiomyopathy.

**Figure 3:**
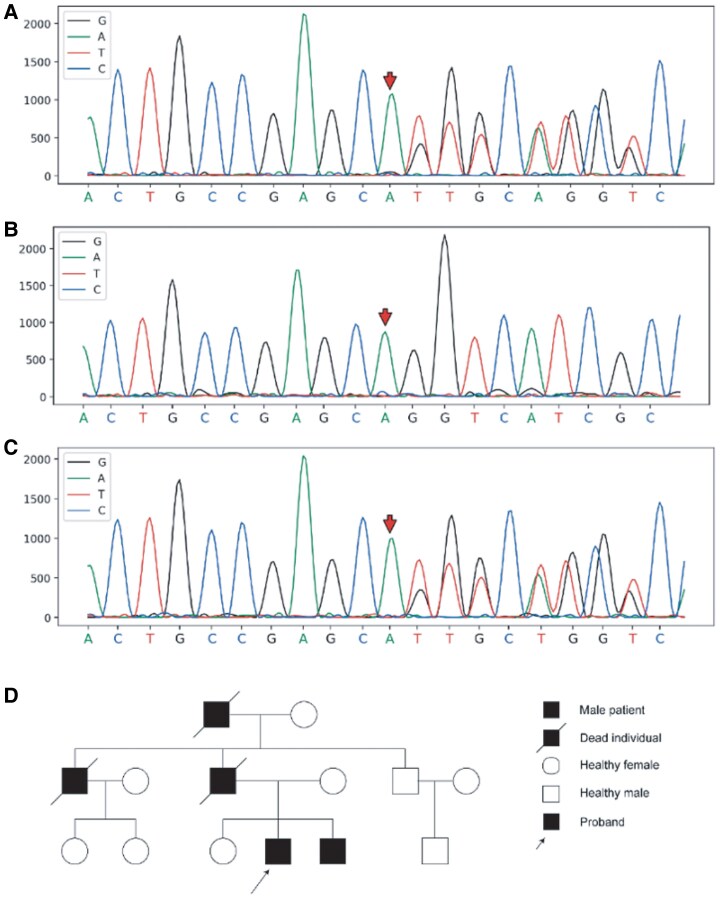
Sanger sequencing and genetic pedigree map. (*A*) Heterozygous mutation at position chr1:236924436 in the proband. (*B*) No mutation was found at position chr1:236924436 in the mother of the proband. (*C*) Heterozygous mutation at position chr1:236924436 in the brother of the proband. (*D*) Genetic pedigree map.

The patient’s cardiomyopathy was strongly suspected to have a genetic origin given the familial clustering of disease manifestations, including the proband’s brother who presented with similar symptoms and the father’s sudden cardiac death at age 35. The family history was further remarkable for premature sudden deaths in paternal relatives, with the paternal uncle dying at age 43 and grandfather at age 59. Whole-exome sequencing of the proband’s blood identified a heterozygous c.2489_2490insTTGCT frameshift mutation in exon 22 of the ACTN2 gene (NM_001103) (*Figure 3*), resulting in a glutamine-to-histidine substitution at position 830 followed by 73 aberrant amino acids before premature termination. This mutation was confirmed in the affected brother but absent in the mother’s analysis, while paternal testing was unfortunately precluded by the father’s death. These findings support an autosomal dominant inheritance pattern of this pathogenic ACTN2 variant associated with the family’s cardiomyopathy phenotype. Further analysis required a blood sample from the patient’s father, who unfortunately had passed away. Despite extensive efforts to contact paternal relatives, additional testing was precluded by the family’s decision to decline further participation.

**Figure 4:**
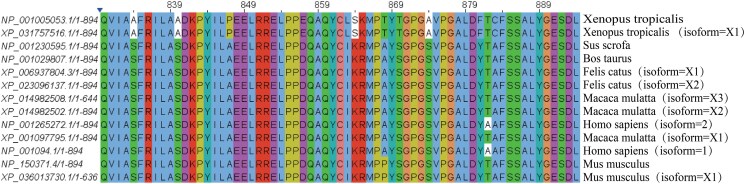
Comparative results of amino acid position 830 and subsequent sequences of ACTN2 gene in different species.

Comparison of the amino acid at position 830 of the ACTN2 gene and subsequent sequences of different species revealed that the sequence is highly conserved among different species, suggesting that the sequence is important for the expression of normal protein function (*Figure 4*).

The ACTN2 c.2489_2490insTTGCT variant is absent in HGMD, ClinVar, and gnomAD East Asian populations, excluding a benign polymorphism. The frameshift causes loss of four HELIX and one STRAND domains in α-actinin-2, likely impairing sarcomeric function. Mutation Taster predicts it as ‘Disease Causing’ (phyloP = 5.187/1.842), consistent with ACMG criteria for pathogenicity (PVS1 + PM1 + PM4 + PP1 + PP3).

## Discussion

ACTN2 gene mutations are associated with significant phenotypic heterogeneity, manifesting across a spectrum of cardiovascular and neuromuscular disorders. Some patients present with dilated cardiomyopathy with ventricular and cardiomyocyte hypertrophy and interstitial fibrosis starting at 7 years of age,^4^ and others present with biventricular hypertrophy and arrhythmias between 10 and 40 years.^5–8^ Additional phenotypes include progressive skeletal myopathy with onset ranging from infancy to adulthood.^9,10^ In this case, the proband’s clinical presentation combined with the identical ACTN2 mutation detected in his affected brother provides compelling evidence for the variant’s pathogenicity.

Current management of restrictive cardiomyopathy remains primarily supportive, focusing on diuretics for volume management, anticoagulation to prevent thromboembolism, and mechanical circulatory support in advanced cases.^1,11^

The persistently poor prognosis of RCM compared to other cardiomyopathies underscores the critical need for novel therapeutic strategies targeting the underlying molecular pathology, as highlighted by this genetically characterized case.

## Data Availability

The data for this case report were taken from the case clinical records and anonymized. Written patient consent was gained for this case report.

## Lead author biography

Dr Bo Lan is a resident doctor at the First Affiliated Hospital of Zhengzhou University in China. He is mainly involved in the diagnosis and treatment of patients with heart failure and cardiomyopathy.

## References

[1] Muchtar E, Blauwet LA, Gertz MA. Restrictive cardiomyopathy: genetics, pathogenesis, clinical manifestations, diagnosis, and therapy. Circ Res 2017;121:819–837. 28912185 10.1161/CIRCRESAHA.117.310982

[2] Brieler J, Breeden MA, Tucker J. Cardiomyopathy: an overview. Am Fam Physician 2017;96:640–646. 29431384

[3] Maron BJ, Towbin JA, Thiene G, Antzelevitch C, Corrado D, Arnett D, et al Contemporary definitions and classification of the cardiomyopathies: an American Heart Association scientific statement from the council on clinical cardiology, heart failure and transplantation committee; quality of care and outcomes research and functional genomics and translational biology interdisciplinary working groups; and council on epidemiology and prevention. Circulation 2006;113:1807–1816. 16567565 10.1161/CIRCULATIONAHA.106.174287

[4] Mohapatra B, Jimenez S, Lin JH, Bowles KR, Coveler KJ, Marx JG, et al Mutations in the muscle LIM protein and alpha-actinin-2 genes in dilated cardiomyopathy and endocardial fibroelastosis. Mol Genet Metab 2003;80:207–215. 14567970 10.1016/s1096-7192(03)00142-2

[5] Theis JL, Bos JM, Bartleson VB, Will ML, Binder J, Vatta M, et al Echocardiographic-determined septal morphology in Z-disc hypertrophic cardiomyopathy. Biochem Biophys Res Commun 2006;351:896–902. 17097056 10.1016/j.bbrc.2006.10.119

[6] Chiu C, Bagnall RD, Ingles J, Yeates L, Kennerson M, Donald JA, et al Mutations in alpha-actinin-2 cause hypertrophic cardiomyopathy: a genome-wide analysis. J Am Coll Cardiol 2010;55:1127–1135. 20022194 10.1016/j.jacc.2009.11.016

[7] Bagnall RD, Molloy LK, Kalman JM, Semsarian C. Exome sequencing identifies a mutation in the ACTN2 gene in a family with idiopathic ventricular fibrillation, left ventricular noncompaction, and sudden death. BMC Med Genet 2014;15:99. 25224718 10.1186/s12881-014-0099-0 PMC4355500

[8] Girolami F, Iascone M, Tomberli B, Bardi S, Benelli M, Marseglia G, et al Novel α-actinin 2 variant associated with familial hypertrophic cardiomyopathy and juvenile atrial arrhythmias: a massively parallel sequencing study. Circ Cardiovasc Genet 2014;7:741–750. 25173926 10.1161/CIRCGENETICS.113.000486

[9] Lornage X, Romero NB, Grosgogeat CA, Malfatti E, Donkervoort S, Marchetti MM, et al ACTN2 mutations cause “multiple structured core disease” (MsCD). Acta Neuropathol 2019;137:501–519. 30701273 10.1007/s00401-019-01963-8 PMC6545377

[10] Savarese M, Palmio J, Poza JJ, Weinberg J, Olive M, Cobo AM, et al Actininopathy: a new muscular dystrophy caused by ACTN2 dominant mutations. Ann Neurol 2019;85:899–906. 30900782 10.1002/ana.25470

[11] Jaquiss RD . Ventricular assistant in restrictive cardiomyopathy: making the right connection. J Thorac Cardiovasc Surg 2016;151:e15–e16. 26395050 10.1016/j.jtcvs.2015.08.085

